# Assessment of COVID-19 Anxiety Levels and Attitudes to COVID-19 Vaccine among Older Adults in Poland: A Pilot Study

**DOI:** 10.3390/vaccines10111918

**Published:** 2022-11-13

**Authors:** Mateusz Cybulski, Zyta Beata Wojszel, Aleksandra Wojszel, Sara Jahel, Paulina Sliwinska, Elzbieta Krajewska-Kulak

**Affiliations:** 1 Department of Integrated Medical Care, Faculty of Health Sciences, Medical University of Bialystok, 15-096 Bialystok, Poland; 2 Department of Geriatrics, Faculty of Health Sciences, Medical University of Bialystok, 15-471 Bialystok, Poland; 3 Department of Geriatrics, Hospital of the Ministry of Interior and Administration, 15-471 Bialystok, Poland; 4 Student’s Scientific Society at the Department of Geriatrics, Faculty of Health Sciences, Medical University of Bialystok, 15-471 Bialystok, Poland

**Keywords:** anxiety, attitudes, COVID-19, fear, older adults, SARS-CoV-2, vaccines, vaccine hesitancy

## Abstract

Background: The fear of being infected with the SARS-CoV-2 has become widespread, especially among older adults. Information campaigns to promote mass vaccination against COVID-19 are a key element in controlling and preventing the spread of the COVID-19 pandemic. However, their success primarily depends on vaccination coverage in a given population. The aim of this study was to assess the severity of COVID-19 anxiety and attitudes towards COVID-19 vaccines among older adults in Poland. Methods: This pilot study was conducted among a total of 127 older participants, including 108 students (85%) of Third Age Universities in Bialystok and 19 patients (15%) of the Department and Clinic of Geriatrics of the Hospital of the Ministry of Internal Affairs and Administration in Bialystok. The study used a diagnostic survey based on an author-designed questionnaire and four standardized psychometric tools: The Fear of COVID-19 Scale (FCV-19S), Coronavirus Anxiety Scale (CAS), The Drivers of COVID-19 Vaccination Acceptance Scale (DrVac-COVID19S), and Scale to Measure the Perception of SARS-CoV-2 Vaccines Acceptance (The VAC-COVID-19 Scale). Results: COVID-19 vaccination coverage in the study group was 88.2%, with three doses in most cases. We found a negative vaccination status only in women taking part in the study. Men scored significantly higher on DrVac-COVID19S and its Value subscale, and markedly lower on FCV-19S. We did not observe significant differences in the scales’ scores between age groups. Respondents recruited from the Third Age Universities had significantly higher scores than geriatric clinic patients in the Knowledge subscale of DrVac-COVID19S. In the case of FCV-19S, no correlation with the results obtained in other scales used in the study was found. Additionally, no correlation was found between CAS scores and the following scales: DrVac-COVID19S (total), DrVac-COVID19S Knowledge (K) subscale, DrVac-COVID19S Autonomy (A) subscale and VAC-COVID-19-Scale-positive subscale. The other scales were strongly correlated with each other—the correlations were statistically significant. Conclusions: Subjective COVID-19 anxiety in the study group was moderate. Seniors were more likely to show positive vaccine attitudes, as confirmed by the percentage of respondents vaccinated against COVID-19 with at least one dose. However, there is still a percentage of unvaccinated individuals in the population of seniors; therefore, measures should be taken to motivate this age group and encourage preventive vaccination against COVID-19. Furthermore, representative studies on COVID-19 anxiety and attitudes towards the COVID-19 vaccine among Polish seniors are needed to determine a more precise prevalence of these phenomena and potential correlations on a national level.

## 1. Introduction

At the end of 2019, a series of cases of pneumonia of unknown etiology was reported in the Chinese province of Wuhan [1]. A few weeks later, an analysis of laboratory samples collected from patients identified a new virus, SARS-CoV-2, responsible for acute respiratory disease [2]. The World Health Organization (WHO) announced “COVID-19” as the name of this new disease. On 11 March 2020, the WHO declared COVID-19 a pandemic [3]. As of 13 October 2022, there have been 623,260,964 confirmed cases of COVID-19 infections globally, including 6,562,631 deaths as a result of the infection [4].

The fear of being infected with SARS-CoV-2 has become widespread. This anxiety is particularly amplified by systematic media messages focusing on epidemiological data on incidence and mortality. The need to maintain social distance, which involves limiting interpersonal contact, is another factor predisposing increased perceived anxiety [5,6,7].

As in many other countries around the world, the Polish population is aging rapidly. The latest demographic data have shown that there are more than 6.7 million (17.5%) adults aged ≥65 in Poland [8]. According to the latest forecasts, this percentage is expected to increase to 25% in 2035, and Poland will become one of the oldest population countries in Europe by 2060, with older people accounting for over one-third of the country’s population [9].

From the very beginning of the pandemic, older adults have been considered to be at the highest risk of SARS-CoV-2 infection and mortality worldwide [10]. The increased risk of morbidity and death depends on several factors. First, Poland is one of the leading countries in Europe in terms of the number of confirmed cases of COVID-19. According to official data, 6,323,108 cases have been diagnosed in Poland since the beginning of the pandemic, including 117,847 deaths, as of 13 October 2022 [11]. Furthermore, multimorbidity, i.e., the coexistence of several chronic diseases, which significantly increases the risk of serious health consequences, also related to COVID-19, is a specific feature of older adults, both in Poland and worldwide [12,13].

Information campaigns to promote mass vaccination against COVID-19 are a key element in controlling and preventing the spread of the pandemic. However, the success of such health education measures depends primarily on vaccination coverage in a given population [14,15]. Older people were the priority group for vaccination worldwide, and the first to receive basic doses as well as booster doses due to their higher risk of developing severe disease if infected. However, the perception of both vaccination and COVID-19 may strongly influence the final decision to vaccinate against COVID-19, and is likely to have a more significant impact than certain sociodemographic characteristics [16,17,18]. The first COVID-19 vaccine in Poland was administered on 27 December 2020. Vaccinations for older adults (70+) began on 25 January 2021. The following vaccines are available in Poland: Pfizer, Moderna, Johnson & Johnson’s, Astra Zeneca, and Novavax. The vast majority of seniors were vaccinated with mRNA vaccines, mainly Pfizer. As of 13 October 2022, 22.57 million people (58.91%) were fully vaccinated in Poland. At least one vaccine dose was received by 75.93% of 60–69-year-olds, 82.14% of 70–79-year-olds, and 66.70% of ≥80+ year-olds [19], which is an extremely disturbing epidemiological trend. Particular attention is drawn to the dramatic decline in the dynamics in making decisions about the need to get vaccinated on the global level. Despite the development and approval of many safe and effective vaccines against COVID-19 in recent months, factors such as varying vaccine efficacy [20], unknown vaccine efficacy against new coronavirus mutants [21], and reported adverse effects of vaccines (e.g., venous thromboembolism (VTE) after AstraZeneca, which is rare compared with the global population) may have had a negative impact on the perception of COVID-19 vaccines [22].

Older adults are a group at particular risk of anxiety due to SARS-CoV-2 and severe COVID-19; therefore, they should have the highest COVID-19 vaccination rates. As such, there is a need to assess the levels of COVID-19 anxiety and attitudes towards vaccination in the geriatric population. Due to the planned nationwide research on these issues, we decided to conduct a pilot study in a convenience sample of active older individuals and geriatric patients, which would allow us to assess the usefulness and reliability of the available scales to assess both the severity of COVID-19 anxiety and attitudes towards COVID-19 vaccination among older adults in Poland. We also assessed the impact of age and gender on the severity of anxiety symptoms and attitudes towards COVID-19 vaccination in the study group of older individuals, as well as we determined the correlations between the tools used.

## 2. Materials and Methods

### 2.1. Participants and Study Design

This pilot study included a total of 127 older people, including 108 auditing students (85%) of Third Age Universities in Bialystok (University of the Third Age, Healthy Senior University, University of Psychogeriatric Prevention) and 19 patients (15%) of the Department and Clinic of Geriatrics of the Hospital of the Ministry of Internal Affairs and Administration in Bialystok.

To this end, our interviewers (medical students, members of the Student Scientific Association at the Department of Geriatrics of the Medical University in Bialystok) reported to all the above-mentioned facilities on the same day and at a pre-specified time (12 May 2022) to conduct a survey in the target group of respondents, who agreed to participate in the study.

The anonymous paper questionnaires were distributed among participants. At the time of completing the questionnaire, the interviewer was at the disposal of the study participants to clarify any doubts. Completed questionnaires were collected and coded for further analysis. The mean time to complete the questionnaire was 12 min.

Age over 60 and written consent to participate in the study were the inclusion criteria for all respondents. The absence of symptoms or diagnosed dementia disorders, confirmed by feedback from the attending physician based on the patient’s medical records, was an additional inclusion criterion for patients treated in the Department/Clinic of Geriatrics. None of the patients were excluded from the study on this basis. Each participant could withdraw from the study at any time.

### 2.2. Measures

The study used the method of a diagnostic survey with the use of an author-designed questionnaire and four standardized psychometric tools: The Fear of COVID-19 Scale (FCV-19S), Coronavirus Anxiety Scale (CAS), The Drivers of COVID-19 Vaccination Acceptance Scale (DrVac-COVID19S), and Scale to Measure the Perception of SARS-CoV-2 Vaccines Acceptance (The VAC-COVID-19 Scale).

The original questionnaire of the survey consisted of 17 questions, including 8 demographics questions. The author-designed short questionnaire enquired about sociodemographic characteristics (gender, age, marital status, education, place of residence, financial status, professional situation, and recruitment group), as well as about a history of SARS-CoV-2 infection and COVID-19 vaccination status. The COVID-19-related questions asked about a history of SARS-CoV-2 infection, confirmed by a positive real-time polymerase chain reaction (RT-PCR) test, and if so, the number of infections. As for SARS-CoV-2-vaccination-related questions, the respondents were asked whether they were vaccinated and, if so, how many doses they received, whether personal or loved one’s exposure to symptomatic SARS-CoV-2 infection had an impact on the decision on whether to vaccinate against COVID-19, and from what sources they obtained information on COVID-19 and SARS-CoV-2 vaccination (multiple-answer question: the respondent could choose from the Internet, television, newspapers, family, friends, medical staff, or named others). Additionally, the respondents were asked about their subjective self-reported mental health before and during the pandemic (closed-ended questions with seven variants of answers from “very poor” to “very good”), and about mental disorders diagnosed by a specialist (open-ended questions).

The Fear of COVID-19 Scale (FCV-19S) was developed to measure anxiety and fear of COVID-19. FCV-19S is a simple seven-item self-administered scale developed by Ahorsu et al. [23]. Answers included “strongly disagree,” “disagree,” “neither agree nor disagree,” “agree,” and “strongly agree.” The minimum score for each question is 1 (strongly disagree), and the maximum is 5 (strongly agree). The total score is calculated by adding up each item’s score (ranging from 7 to 35). The higher the score, the greater the fear of COVID-19 [24]. FCV-19S has been translated and validated in several countries, including Poland [25].

The Coronavirus Anxiety Scale (CAS) is a brief self-reported mental health screener of dysfunctional anxiety associated with the COVID-19 crisis, which consists of five items related to various physical and mental ailments that appear in response to news or thoughts about COVID-19. Each item contains answers from 0 (“not at all”) to 4 (“nearly every day over the last two weeks”) [26]. Cronbach’s α and McDonald’s ω coefficients for the Polish version of CAS were α = 0.93 and ω = 0.93, respectively, assessed in a group of front-line healthcare workers [27].

The VAC-COVID-19 scale is a valid and reliable instrument of public health to measure the perception of SARS-CoV-2 vaccine acceptance. This scale can be useful to determine why different populations do or do not adhere to the vaccination to help propose adequate and effective strategies to advance vaccination coverage rates. The VAC-COVID-19 scale is a simple eleven-item self-administered scale developed by Mejia et al. [28]. There are two groups of factors: positive (reasons for receiving vaccination) and negative (reasons for not receiving vaccination). Each item had five possible Likert-type responses: strongly disagree, disagree, neither disagree nor agree, agree, and strongly agree. The minimum score for each question is 1 (strongly disagree), and the maximum is 5 (strongly agree). Reverse scoring applies to the second (negative) group of factors. The total score is calculated by adding up each item’s score (ranging from 11 to 55). The higher the score, the more positive the attitudes towards COVID-19 vaccinations. The Cronbach’s α coefficient for this tool was α = 0.831 [28].

The DrVac-COVID19S was adapted from the MoVac-Flu Scale [29]. The major difference between DrVac-COVID19S and MoVac-Flu Scale is that MoVac-Flu Scale uses the word flu, and DrVac-COVID19S uses the term COVID-19. DrVac-COVID19S contains 12 items, where nine items are positively worded (items 1 to 6, 8, 9, and 12), and three items are negatively worded (items 7, 10, and 11). Therefore, DrVac-COVID19S shares the same model of CME as the MoVac-Flu Scale in assessing an individual’s values, impacts, knowledge, and autonomy traits. The four traits can help healthcare providers and researchers to understand how an individual (i) cares about the purpose of COVID-19 vaccination uptake (values); (ii) believes in the effects of COVID-19 vaccination uptake in preventing COVID-19 infection (impacts); (iii) possesses COVID-19 vaccination uptake (knowledge); and (iv) is confident and has control in receiving a COVID-19 vaccination if they want to (autonomy). Moreover, the 12 items comprise four traits corresponding to the CME model: items 3 (“It is important that I get the COVID-19 jab”), 6 (“The COVID-19 jab plays an important role in protecting my life and that of others”), and 8 (“The contribution of the COVID-19 jab to my health and well-being is very important”) comprise values; items 1 (“Vaccination is a very effective way to protect me against the COVID-19”), 4 (“Vaccination greatly reduces my risk of catching COVID-19), and 12 (“Getting the COVID-19 jab has a positive influence on my health”) comprise impacts; items 2 (“I know very well how vaccination protects me from the COVID-19”), 5 (“I understand how the flu jab helps my body fight the COVID-19 virus”), and 10 (“How the COVID-19 jab works to protect my health is a mystery to me”) comprise knowledge; and items 7 (“I feel under pressure to get the COVID-19 jab”), 9 (“I can choose whether to get a COVID-19 jab or not”), and 11 (“I get the COVID-19 jab only because I am required to do so”) comprise autonomy. All the items are rated using a seven-point Likert scale. After reverse coding the negatively worded items (i.e., scoring for these items from 1 (strongly agree) to 7 (strongly disagree)), a higher score in the DrVac-COVID19S indicated a higher level of COVID-19 vaccine acceptance [29].

### 2.3. Procedure and Ethical Considerations 

This study was conducted following the recommendations and was reviewed and approved by the Bioethics Committee of the Medical University in Bialystok (statute no. APK.002.436.2021). All subjects gave written informed consent, in accordance with the Declaration of Helsinki.

### 2.4. Statistical Analysis 

The IBM SPSS Version 18 Software suit (SPSS, Chicago, IL, USA) was used to analyze the data collected. Descriptive statistics were expressed as the mean (M) and standard deviation (SD) or median (Me) and interquartile range (IQR), as appropriate, for continuous variables, and as the minimum (Min), maximum (Max), kurtosis (K), and skewness (SK) in the case of the scales used in the study. The Shapiro–Wilk test was used to assess the normality of the distribution of the quantitative variables. Categorical variables were expressed as the frequency (N) and percentage (%). As appropriate, differences between groups (such as age and sex categories) were expressed using the chi-square χ^2^, the Mann–Whitney U test, or the Student’s *t*-test. Cronbach’s alpha and McDonald’s omega were used to assess the internal consistency of the scales, and Spearman’s rank correlation coefficient was used to evaluate correlations between quantitative variables. McNemar’s change test for dependent samples was used to determine if there were differences in dichotomous dependent variables between two related groups. The results were considered statistically significant at *p* < 0.05.

## 3. Results

### 3.1. Sample Characteristics

In total, 127 respondents took part in the study. The median age was 70 years (IQR, 67–75), with most participants younger than 75 years old; 85% were women. Table 1 presents the sociodemographic characteristics of the respondents as well as their mental disorders and self-reported mental health before and during the pandemic, SARS-CoV-2 infection and vaccination status, and factors that could influence their decision to be vaccinated.

The respondents recruited from Third Age Universities were significantly younger, predominantly <75 years old (81.8% vs. 31.6% in geriatric clinics patients). Additionally, most women were under 75 years of age (78.7% vs.47.4% in men, *p* = 0.004).

The educational status of respondents aged 75+ was significantly worse, and they had significantly worse self-reported mental health before and during the pandemic than their younger counterparts. A similar percentage of younger and older respondents had mental disorders before the pandemic, with older patients reporting such history significantly more frequently during the pandemic (most often depression and anxiety disorders). One-third of respondents had had previous SARS-CoV-2 infection (40 persons once, and four persons twice during the pandemic). 

COVID-19 vaccination coverage in the study group was 88.2%, with three doses in most cases. We only observed a negative vaccination status in women taking part in the study. The respondents learnt about COVID-19 and vaccinations mainly from the internet (significantly more often younger respondents—77.7% vs. 48.5% in 75+, *p* = 0.002) and television, and less frequently from medical staff, friends, press, family, or other sources (*n* = 3 (2.4%)—radio; *n* = 1 (0.8%)—own observations). Respondents aged ≥75 years significantly more often reported family as the main source of information on that topic (42.4% vs. 21.3% in respondents <75 years of age, *p* = 0.02).

The occurrence of mental disorders during the pandemic was reported significantly more often (15.7% vs. 4.7% before the pandemic, *p* = 0.021).

### 3.2. Scales and Descriptive Statistics

In the CAS, respondents were asked to choose an answer that best described how they had felt and conducted themselves over the past two weeks (Table 2). Significantly more respondents aged ≥75 years reported feeling dizzy, lightheaded, or faint when they read or listened to news about COVID-19. In addition, women significantly more often had trouble falling or staying asleep because they thought about the coronavirus. No other differences between age or sex groups were noted.

The answer “I am most afraid of coronavirus” in FCV-19S (Table 3) was given significantly more often by women (*p* = 0.049). In addition, younger respondents significantly more frequently said that thinking about coronavirus made them uncomfortable (*p* = 0.026); less often than respondents 75+ years of age, they reported that their hands became sweaty when they thought about coronavirus (*p* = 0.024). We did not observe any other significant differences between age and sex groups.

There were no significant differences in the frequency of response to particular questions on DrVac-COVID19S (Table 4) between the sex or age groups. In the case of “I can choose whether to get a COVID-19 jab or not,” affirmative answers were given significantly more often by younger participants (under the age of 75 years) (*p* = 0.013).

Table 5 presents the distribution of the answers to the questions in the VAC-COVID-19 Scale. Younger respondents under the age of 75 years significantly more often agreed with the statement that SARS-CoV-2 vaccines were part of the plan of a large company that had created COVID-19 than their older counterparts (*p* = 0.022).

Table 6 shows the descriptive statistics of the psychometric scales used in the study. Both Cronbach’s alpha and McDonald’s omega confirmed the high internal consistency of the majority of scales, apart from the Knowledge and Autonomy subscales of the DrVac-COVID19S scale. However, the scale showed excellent reliability (both indices were >0.9 in this case).

### 3.3. Scales Answers and Descriptive Statistics

Table 7 shows the results of the psychometric scales used in the study for the sex groups. Men scored significantly more highly on DrVac-COVID19S and its Value subscale and markedly lower on FCV-19S.

We found no significant differences in the scores between age groups. Respondents recruited from Third Age Universities had significantly higher scores than geriatric clinic patients in the Knowledge subscale of the DrVac-COVID19 scale (Me-15, IQR 12–18 vs. Me = 11, IQR 8–13; *p* < 0.001), although the groups did not differ in the results of other scales used in the study.

### 3.4. Correlations between Psychometric Scale Results

Table 8 shows Spearman’s rank correlations between standardized psychometric scales used in the study. In the case of the results obtained in FCV-19S, no correlation with the results obtained in other scales used in the study was found. Additionally, no correlation was found between the CAS scores and the scores in the following scales: DrVac-COVID19S (total), DrVac-COVID19S Knowledge (K) subscale, DrVac-COVID19S Autonomy (A) subscale and VAC-COVID-19-Scale-positive subscale. The other scales strongly correlated with each other, and the correlations were statistically significant.

## 4. Discussion

### 4.1. Fear of COVID-19

Our study showed a moderate level of COVID-19 anxiety in the study population of older individuals (6.52 ± 2.19 for CAS and 17.67 ± 6.11 for FCV-19S). These results were lower than those obtained in our previous research, in which non-specific tools were used to measure COVID-19 anxiety (General Anxiety Disorder-7 (GAD-7), Short Health Anxiety Inventory (SHAI), and State-Trait Anxiety Inventory (STAI)) [30]. Agrawal et al. [31], who included 500 older individuals from Wroclaw (Poland) in their study, showed a mean FCV-19S anxiety score of 19.3 ± 5.6. Overall, this may suggest that the health situation of Polish seniors in terms of corona-phobia is beginning to improve, and that this group has adapted to the COVID-19 pandemic. Higher mean FCV-19S scores were reported in international studies. Mistry et al. [32] showed that the COVID-19 pandemic caused considerable fear among the older Bangladeshi population, with a mean score of 19.4 ± 6.1.

Our study showed that the level of fear of SARS-CoV-2 infection is higher among women than men. Our results are consistent with other, previously published data, which describe gender differences in pandemic-induced behaviors [31,33,34,35]. The relationship between COVID-19 anxiety and gender was described by Hosen et al. [36]. The authors showed that men exhibited more risky health behaviors in the face of the COVID-19 pandemic, indicating lower health awareness about the possibility of SARS-CoV-2 infection. On the other hand, women were more likely to adapt to the current recommendations, including those on compliance with the sanitary regime, which is related to the higher female health awareness about COVID-19, and thus may cause stressful situations, in particular regarding their immediate family, thereby increasing anxiety about contracting severe COVID-19.

At the same time, our study showed no statistically significant relationship between age and the fear of developing COVID-19 in CAS, whereas a reverse, statistically significant correlation was found for FCV-19S. Both Polish [30,37] and foreign [38,39,40] publications showing that age is negatively correlated with anxiety symptoms during a pandemic, as well as papers confirming high levels of anxiety among people in the oldest age groups, especially in China [41,42], can be found in the current literature.

In our research, a significant percentage of respondents agreed with FCV-19S statements regarding concerns related to COVID-19: 38.6% of respondents felt uncomfortable thinking about the coronavirus, 42.5% of respondents were afraid of being infected with SARS-CoV-2, and 47.3% of respondents were afraid of losing their lives due to COVID-19.

The results of our research are consistent with those available in the international literature [32,43,44]. Chalhoub et al. [43], who conducted their research in a Lebanese population, showed that 41.9% of respondents felt uncomfortable thinking about the new coronavirus, 33.7% were afraid of COVID-19, and 23.8% were afraid of losing their lives due to the disease. Furthermore, 35.4% of participants in the same study felt nervous or upset when exposed to COVID-19 news on TV and social media. This proportion was higher (39.3%) in our research.

### 4.2. Attitudes to COVID-19 Vaccines

Low acceptance of COVID-19 vaccines in the general population may contribute to continuous epidemics, as well as intensify the challenges related to controlling the spread of SARS-CoV-2 both in Poland and worldwide; therefore, it is extremely important to define the beliefs about COVID-19 vaccination among older adults, who are at a particular risk of severe infection. A longitudinal U.S. study conducted in a group of middle-aged and older people showed that the vast majority supported preventive vaccinations and had received the COVID-19 vaccine by May 2021 [45]. Our study also confirmed mostly positive attitudes towards COVID-19 vaccines, as evidenced by the mean DrVac-COVID19S and VAC-COVID-19 scores.

In the study group, 88.2% of seniors were vaccinated, including 87.4% fully vaccinated (at least two doses). In a Brazilian study [46], which assessed COVID-19 vaccine acceptance among older adults (no vaccines were available at the time of the study), 91.8% of respondents declared their readiness to get vaccinated. The cited study showed one of the highest COVID-19 vaccine acceptance rates compared with studies conducted in other countries, especially in high-income countries [16,47]. Similar results were obtained by American researchers in a study conducted among individuals aged ≥65 years in November 2020. The vast majority of respondents (91%) reported COVID-19 vaccine acceptance. However, even in the older population with adequate health awareness and confidence in the health care system, nearly one in ten respondents were not interested in receiving the vaccine [48]. In our study, an average of one in eight respondents (11.8%) were not vaccinated. However, according to Al-Hanawi et al. in Saudi Arabia [49], more than one-half of older adults (56.14%) reported that they refuse to be vaccinated against COVID-19. The high rate of vaccine refusal may be partly due to the widespread fake news and conspiracy theories about vaccine safety and efficacy. Such information can cause fear and raise doubts about the origin and safety of vaccines, and consequently pose threats to the massive vaccination campaign [50]. Therefore, measures should be taken to increase the emphasis of the need to maximize the public acceptance of vaccination, particularly in the oldest age groups.

In our study, we found lower rates of reported vaccine acceptance in women compared with men, as also shown in many other studies [16,18,48,49,51,52]. This may be due to the gender differences in adverse events and vaccine-induced humoral immune responses among women, as well as a higher risk of COVID-19 complications, mortality, and higher infectivity among men [53,54,55]. At this point, however, it is worth mentioning that because our study only included 19 men, accounting for 15% of the study group, the results are not sufficiently representative to be fully comparable with the cited research.

### 4.3. Limitations

Our study has some limitations. First, the presented results come from a study based on a subjective assessment of anxiety symptoms in older adults. Standardized scales were used in the study, which are sensitive research tools, but are based on subjective feelings rather than objective criteria of clinical symptoms, which may result in false-positive results. Secondly, because this was a pilot study, the sample was too small to generalize the results to the entire population of Polish seniors. Thirdly, the study group was over-represented by women; hence, the results should be verified in an equally numerous group of men. However, the actual demographic trends in the Polish population are characterized by a high percentage of women compared with men (over-representation of senior women in relation to men), which is a significant limitation. Fourthly, the selection of study subgroups may also be a limitation in subsequent studies. Geriatric patients are very often characterized by multimorbidity and polypharmacy, which may increase the fear of COVID-19 and be a factor encouraging vaccination against COVID-19. On the other hand, seniors attending Universities of the Third Age have more awareness and knowledge about COVID-19 and vaccines then standard geriatric patients. Despite these limitations, our results may be a starting point for further research on COVID-19 anxiety attitudes to vaccination against COVID-19 among older adults in Poland and their sociodemographic determinants. Ideally, these questions should be addressed by a nationwide longitudinal study.

## 5. Conclusions

1. The subjective COVID-19 anxiety in the study group was at a moderate level, which was lower compared with studies available in the current literature.
2. Seniors were more likely to show vaccine acceptance attitudes, as confirmed by the percentage of respondents vaccinated with at least one dose.
3. There is still a percentage of unvaccinated people in the senior population; therefore, measures should be taken to motivate this age group and to encourage vaccination against COVID-19.
4. Furthermore, representative studies on COVID-19 anxiety and attitudes towards COVID-19 vaccination among Polish seniors are needed for the more precise determination of the prevalence of these phenomena and potential correlations on a national level.

## Figures and Tables

**Table 1:** Sociodemographic characteristics of the respondents.

	Sociodemographic Feature	*n*	%
Sex	women	108	85.0
	men	19	15.0
Age	<75 years	94	74.0
	75+ years	33	26.0
Marital status	married	70	55.1
	separation	1	0.8
	divorced	11	8.7
	widowed	39	30.7
Education	higher	74	58.3
	secondary	44	34.6
	primary	7	5.5
	lack of education	2	1.6
Place of residence	village	12	9.4
	town up to 50,000	32	25.2
	town up to 100,000	12	9.4
	city up to 300,000	54	42.5
	city over 300,000	17	13.4
Financial status	very good	7	5.5
	good	57	44.9
	rather good	31	24.4
	average	29	22.8
	rather poor	3	2.4
Socio-professional status	retirement pension	116	91.3
	disability pension	2	1.6
	professionally active	9	7.1
Recruitment group	Third Age Universities	108	85.0
	out/in-patient geriatric departments	19	15.0
Self-reported mental health before the pandemic	very poor	1	0.8
	poor	1	0.8
	rather poor	3	2.4
	not poor, not good	4	6.3
	rather good	21	16.5
	good	59	46.5
	very good	34	26.8
History of mental disorders before the pandemic	yes	12	9.4
	no	115	90.6
Mental disorders diagnosed before the pandemic	depression	6	4.7
	anxiety	4	3.1
	neurosis	1	0.8
	social withdrawal	1	0.8
Self-reported mental health during the pandemic	very poor	2	1.6
	poor	6	4.7
	rather poor	16	12.6
	nor poor, nor good	24	18.9
	rather good	33	26.0
	good	38	29.9
History of mental disorders during the pandemic	yes	20	15.7
	no	107	84.3
Mental disorders diagnosed during the pandemic	depression	10	7.9
	anxiety	7	5.5
	neurosis	1	0.8
	social withdrawal	1	0.8
	loneliness	1	0.8
Previous SARS-CoV-2 infection (RT-PCR confirmed)	yes	44	34.6
	no	83	65.4
Vaccination status	unvaccinated	15	11.8
	vaccinated with one dose	1	0.8
	vaccinated with two doses	16	12.6
	vaccinated with three doses	94	74.0
	vaccinated with four doses	1	0.8
Learned about COVID-19 and vaccinations from:	Internet	89	70.1
	television	84	66.1
	newspapers	36	28.3
	family	34	26.8
	friends	41	32.3
	medical staff	46	36.2
	other sources (radio, own observations)	4	3.1
A close person’s history of SARS-CoV-2 infection influenced the decision to vaccinate	yes	18	14.2
	no	109	85.8
What role did the family/friends play in deciding about vaccination?	They encouraged vaccination	77	60.6
	They discouraged vaccination	8	6.3
	It is difficult to say	42	33.1

**Table 2:** Coronavirus Anxiety Scale (CAS).

Item	Answers (Scores)	*n* = 127	%
I felt dizzy, lightheaded, or faint when I read or listened to news about the coronavirus.	Not at all (1)	114	89.9
	Rare, less than a day or two (2)	9	7.1
	Several days (3)	4	3.1
	More than 7 days (4)	-	-
	Nearly every day over the last 2 weeks (5)	-	-
I had trouble falling or staying asleep because I was thinking about the coronavirus.	Not at all (1)	84	66.1
	Rare, less than a day or two (2)	29	22.8
	Several days (3)	11	8.7
	More than 7 days (4)	1	0.8
	Nearly every day over the last 2 weeks (5)	2	1.6
I felt paralyzed or frozen when I thought about or was exposed to information about the coronavirus.	Not at all (1)	76	59.8
	Rare, less than a day or two (2)	35	27.6
	Several days (3)	14	11.0
	More than 7 days (4)	-	-
	Nearly every day over the last 2 weeks (5)	2	1.6
I lost interest in eating when I thought about or was exposed to information about the coronavirus.	Not at all (1)	110	86.6
	Rare, less than a day or two (2)	16	12.6
	Several days (3)	1	0.8
	More than 7 days (4)	-	-
	Nearly every day over the last 2 weeks (5)	-	-
I felt nauseous or had stomach problems when I thought about or was exposed to information about the coronavirus.	Not at all (1)	105	82.7
	Rare, less than a day or two (2)	19	15.0
	Several days (3)	3	2.4
	More than 7 days (4)	-	-
	Nearly every day over the last 2 weeks (5)	-	-

**Table 3:** The Fear of COVID-19 Scale (FCV-19S).

Item	Answers (Scores)	*n* = 127	%
I am most afraid of coronavirus.	Strongly disagree (1)	17	13.4
	Disagree (2)	23	18.1
	Partly disagree/partly agree (3)	33	26.0
	Agree (4)	39	30.7
	Strongly agree (5)	15	11.8
It makes me uncomfortable to think about coronavirus.	Strongly disagree (1)	18	14.2
	Disagree (2)	22	17.3
	Partly disagree/partly agree (3)	38	29.9
	Agree (4)	33	26.0
	Strongly agree (5)	16	12.6
My hands become clammy when I think about coronavirus.	Strongly disagree (1)	62	48.8
	Disagree (2)	53	41.7
	Partly disagree/partly agree (3)	9	7.1
	Agree (4)	3	2.4
	Strongly agree (5)	-	-
I am afraid of losing my life because of coronavirus.	Strongly disagree (1)	28	22.0
	Disagree (2)	18	14.2
	Partly disagree/partly agree (3)	21	16.5
	Agree (4)	42	33.1
	Strongly agree (5)	18	14.2
When watching news and stories about coronavirus on social media I become nervous or anxious.	Strongly disagree (1)	22	17.3
	Disagree (2)	23	18.1
	Partly disagree/partly agree (3)	32	25.2
	Agree (4)	36	28.3
	Strongly agree (5)	14	11.0
I cannot sleep because I’m worrying about getting coronavirus.	Strongly disagree (1)	56	44.1
	Disagree (2)	40	31.5
	Partly disagree/partly agree (3)	21	16.5
	Agree (4)	7	5.5
	Strongly agree (5)	3	2.4
My heart races or palpitates when I think about getting coronavirus.	Strongly disagree (1)	56	44.1
	Disagree (2)	35	27.6
	Partly disagree/partly agree (3)	22	17.3
	Agree (4)	11	8.7
	Strongly agree (5)	3	2.4

**Table 4:** The Drivers of COVID-19 Vaccination Acceptance Scale (DrVac-COVID19S).

Item	Answers (Scores)	*n* = 127	%
1. Vaccination is a very effective way to protect me against COVID-19. (I)	Strongly disagree (1)	9	7.1
	Disagree (2)	4	3.1
	Slightly disagree (3)	8	6.3
	Neither disagree nor agree (4)	7	5.5
	Slightly agree (5)	19	15.0
	Agree (6)	29	22.8
	Strongly agree (7)	51	40.2
2. I know very well how vaccination protects me from COVID-19. (K)	Strongly disagree (1)	11	8.7
	Disagree (2)	6	4.7
	Slightly disagree (3)	8	6.3
	Neither disagree nor agree (4)	8	6.3
	Slightly agree (5)	21	16.5
	Agree (6)	31	24.4
	Strongly agree (7)	42	33.1
3. It is important that I get the COVID-19 jab. (V)	Strongly disagree (1)	9	7.1
	Disagree (2)	4	3.1
	Slightly disagree (3)	3	2.4
	Neither disagree nor agree (4)	4	3.1
	Slightly agree (5)	21	16.5
	Agree (6)	32	25.2
	Strongly agree (7)	54	42.5
4. Vaccination greatly reduces my risk of catching COVID-19. (I)	Strongly disagree (1)	8	6.3
	Disagree (2)	5	3.9
	Slightly disagree (3)	9	7.1
	Neither disagree nor agree (4)	2	1.6
	Slightly agree (5)	16	12.6
	Agree (6)	31	24.4
	Strongly agree (7)	56	44.1
5. I understand how the flu jab helps my body fight the COVID-19 virus. (K)	Strongly disagree (1)	11	8.7
	Disagree (2)	9	7.1
	Slightly disagree (3)	3	2.4
	Neither disagree nor agree (4)	25	19.7
	Slightly agree (5)	16	12.6
	Agree (6)	34	26.8
	Strongly agree (7)	29	22.8
6. The COVID-19 jab plays an important role in protecting my life and that of others. (V)	Strongly disagree (1)	9	7.1
	Disagree (2)	6	4.7
	Slightly disagree (3)	3	2.4
	Neither disagree nor agree (4)	6	4.7
	Slightly agree (5)	16	12.6
	Agree (6)	33	26.0
	Strongly agree (7)	54	42.5
7. I feel under pressure to get the COVID-19 jab. (A)	Strongly disagree (7)	51	40.2
	Disagree (6)	35	27.6
	Slightly disagree (5)	12	9.4
	Neither disagree nor agree (4)	7	5.5
	Slightly agree (3)	9	7.1
	Agree (2)	5	3.9
	Strongly agree (1)	8	6.3
8. The contribution of the COVID-19 jab to my health and well-being is very important. (V)	Strongly disagree (1)	10	7.9
	Disagree (2)	7	5.5
	Slightly disagree (3)	6	4.7
	Neither disagree nor agree (4)	13	10.2
	Slightly agree (5)	12	9.4
	Agree (6)	35	27.6
	Strongly agree (7)	44	34.6
9. I can choose whether to get a COVID-19 jab or not. (A)	Strongly disagree (1)	6	4.7
	Disagree (2)	2	1.6
	Slightly disagree (3)	6	4.7
	Neither disagree nor agree (4)	3	2.4
	Slightly agree (5)	12	9.4
	Agree (6)	38	29.9
	Strongly agree (7)	60	47.2
10. How the COVID-19 jab works to protect my health is a mystery to me. (K)	Strongly disagree (7)	25	19.7
	Disagree (6)	21	16.5
	Slightly disagree (5)	13	10.2
	Neither disagree nor agree (4)	14	11.0
	Slightly agree (3)	17	13.4
	Agree (2)	21	16.5
	Strongly agree (1)	16	12.6
11. I get the COVID-19 jab only because I am required to do so. (A)	Strongly disagree (7)	56	44.1
	Disagree (6)	32	25.2
	Slightly disagree (5)	10	7.9
	Neither disagree nor agree (4)	6	4.7
	Slightly agree (3)	7	5.5
	Agree (2)	6	4.7
	Strongly agree (1)	10	7.9
12. Getting the COVID-19 jab has a positive influence on my health. (I)	Strongly disagree (1)	10	7.9
	Disagree (2)	7	5.5
	Slightly disagree (3)	4	3.1
	Neither disagree nor agree (4)	12	9.4
	Slightly agree (5)	14	11.0
	Agree (6)	36	38.3
	Strongly agree (7)	44	34.6

Abbreviations: A—autonomy subscale; I—influence subscale; K—knowledge subscale; V—values subscale.

**Table 5:** Scale to Measure the Perception of SARS-CoV-2 Vaccines Acceptance (The VAC-COVID-19 Scale).

Item	Answers (Scores)	*n* = 127	%
Negative factor: I should not get SARS-CoV-2 vaccines because...			
1. I think they are going to insert electronic chips/transistors to control my brain.	Strongly agree (1)	4	3.1
	Agree (2)	2	1.6
	Partly disagree/partly agree (3)	9	7.1
	Disagree (4)	4	3.1
	Strongly disagree (5)	108	85.0
2. I think SARS-CoV-2 vaccines are part of the plan of a large company that created COVID-19.	Strongly agree (1)	4	3.1
	Agree (2)	11	8.7
	Partly disagree/partly agree (3)	9	7.1
	Disagree (4)	14	11.0
	Strongly disagree (5)	89	70.1
3. I think that some SARS-CoV-2 vaccines can come from a former communist republic (like Russia), which may result in influences on communist thinking.	Strongly agree (1)	2	1.6
	Agree (2)	1	0.8
	Partly disagree/partly agree (3)	8	6.3
	Disagree (4)	9	7.1
	Strongly disagree (5)	107	84.3
4. I think COVID-19 is an invention of the World Health Organization (WHO) or other similar institutions.	Strongly agree (1)	5	3.9
	Agree (2)	9	7.1
	Partly disagree/partly agree (3)	15	11.8
	Disagree (4)	16	12.6
	Strongly disagree (5)	82	64.6
5. I think COVID-19 does not exist. It is an invention.	Strongly agree (1)	5	3.9
	Agree (2)	-	-
	Partly disagree/partly agree (3)	10	7.9
	Disagree (4)	9	7.1
	Strongly disagree (5)	103	81.1
6. A healthy life is enough to fight disease.	Strongly agree (1)	8	6.3
	Agree (2)	14	11.0
	Partly disagree/partly agree (3)	8	6.3
	Disagree (4)	42	33.1
	Strongly disagree (5)	55	43.3
7. I do not trust in my health care system (including health care personnel).	Strongly agree (1)	6	4.7
	Agree (2)	4	3.1
	Partly disagree/partly agree (3)	25	19.7
	Disagree (4)	23	18.1
	Strongly disagree (5)	69	54.3
Positive factor: I should get SARS-CoV-2 vaccines because...			
8. I want to get back to the life I had before the pandemic.	Strongly agree (5)	75	59.1
	Agree (4)	30	23.6
	Partly disagree/partly agree (3)	5	3.9
	Disagree (2)	3	2.4
	Strongly disagree (1)	14	11.0
9. SARS-CoV-2 vaccines should contribute to improving the health of my family or loved ones.	Strongly agree (5)	59	46.5
	Agree (4)	35	27.6
	Partly disagree/partly agree (3)	10	7.9
	Disagree (2)	8	6.3
	Strongly disagree (1)	15	11.8
10. I think SARS-CoV-2 vaccines should contribute to improving the health of the community/population.	Strongly agree (5)	62	48.8
	Agree (4)	34	26.8
	Partly disagree/partly agree (3)	10	7.9
	Disagree (2)	7	5.5
	Strongly disagree (1)	14	11.0
11. I do not want to wear personal protective equipment anymore (masks).	Strongly agree (5)	49	38.6
	Agree (4)	37	29.1
	Partly disagree/partly agree (3)	18	14.2
	Disagree (2)	9	7.1
	Strongly disagree (1)	14	11.0

**Table 6:** Descriptive statistics for the standardized psychometric scales used in the study.

Scale	M	SD	Me	IQR	Min	Max	SKE	K	Cronbach’s α	McDonald’s ω
CAS	6.52	2.19	5.0	5–7	5.0	15.0	1.72	2.68	0.765	0.784
FCV-19S	17.67	6.11	17.0	14–22	7.0	32.0	0.12	−0.51	0.876	0.881
DrVac-COVID19S-total	64.19	15.95	69.0	56–75	21.0	84.0	−1.003	0.24	0.912	0.916
Values subscale	16.53	5.17	18	15–21	3	21	−1.37	1.02	0.933	0.936
Impacts subscale	16.92	3.85	18	14–20	5	21	−0.901	0.04	0.957	0.957
Knowledge subscale	14.33	4.53	15	11–18	3	21	−0.51	−0.25	0.646	0.667
Autonomy subscale	13.88	2.57	14	13–15	3	21	−0.62	2.56	0.527	0.698
VAC-COVID-19-Scale-negative subscale	30.72	5.18	33	28–35	12.0	35.0	−1.43	1.47	0.832	0.834
VAC-COVID-19-Scale-positive subscale	15.82	4.30	17	14–19	4	20	−1.26	1.04	0.819	0.831
VAC-COVID-19-Scale-total score	46.54	8.18	49	42–52	21	55	−1.24	1.03	0.856	0.848

Abbreviations: CAS—Coronavirus Anxiety Scale; DrVac-COVID19S—The Drivers of COVID-19 Vaccination Acceptance Scale; FCV-19S—The Fear of COVID-19 Scale; M—medium; Me—median; IQR—interquartile range; K—kurtosis; SD—standard deviation; SKE—skewness; VAC-COVID-19—Scale to Measure the Perception of SARS-CoV-2 Vaccines Acceptance.

**Table 7:** Impact of sex on the scores obtained in the psychometric scales used in the study.

Scale	Women (*n* = 108)		Men (*n* = 19)		*p*
	M ± SD	Me (IQR)	M ± SD	Me (IQR)	
CAS	6.59 ± 2.12	6 (5–7)	6.11 ± 2.54	5 (5–6)	0.057 ^a^
FCV-19S	18 ± 6.07	18 (15–22)	14.47 ± 5.42	15 (9–18)	0.013 ^b,^*
DrVac-COVID19S	63.09 ± 16.16	68.5 (54–74)	70.42 ± 13.43	73 (66–77)	0.035 ^a,^*
Values subscale	16.17 ± 5.27	18 (14–21)	18.58 ± 4.10	20 (18–21)	0.048 ^a,^*
Impacts subscale	16.86 ± 3.94	18 (14–20)	17.26 ± 3.31	18 (15–21)	0.854 ^a^
Knowledge subscale	14.08 ± 4.59	15 (11.17.5)	15.74 ± 3.98	16 (15–18)	0.126 ^a^
Autonomy subscale	13.99 ± 2.35	14 (13–15)	13.26 ± 3.60	14 (12–15)	0.582 ^a^
VAC-COVID-19-Scale-negative subscale	30.45 ± 5.25	32 (28–35)	32.21 ± 4.63	34 (33–35)	0.098 ^a^
VAC-COVID-19-Scale-positive subscale	15.79 ± 4.39	17 (14–19.75)	16.0 ± 3.8	16 (14–19)	0.935 ^a^
VAC-COVID-19-Scale-total score	46.24 ± 8.47	48 (42.25–52)	48.21 ± 6.17	51 (42–53)	0.539 ^a^

Abbreviations: ^a^—Mann–Whitney’s test; ^b^—*t*-Student’s test; CAS—Coronavirus Anxiety Scale; DrVac-COVID19S—The Drivers of COVID-19 Vaccination Acceptance Scale; FCV-19S—The Fear of COVID-19 Scale; M—medium; Me—median; IQR—interquartile range; *p*—*p*-value; SD—standard deviation; VAC-COVID-19—Scale to Measure the Perception of SARS-CoV-2 Vaccines Acceptance, and *—statistically significant.

**Table 8:** Spearman’s rank correlations between standardized psychometric scales used in the study.

Scale		CAS	FCV-19S	DrVac-COVID19S	V	I	K	A	VAC-COVID-19-Scale-Negative	VAC-COVID-19-Scale-Positive
FCV-19S	r	0.426								
	*p*	<0.001 *								
DrVac-COVID19S	r	−0.147	−0.042							
	*p*	0.098	0.636							
Values (V) subscale	r	−0.181	0.052	0.892						
	*p*	0.042 *	0.562	<0.001 *						
Impacts (I) subscale	r	−0.213	−0.062	0.666	0.561					
	*p*	0.016 *	0.490	<0.001 *	<0.001 *					
Knowledge (K) subscale	r	0.007	−0.071	0.820	0.594	0.317				
	*p*	0.934	0.427	<0.001 *	<0.001 *	<0.001 *				
Autonomy (A) subscale	r	−0.147	0.173	0.412	0.507	0.402	0.181			
	*p*	0.100	0.052	<0.001 *	<0.001 *	<0.001 *	0.042 *			
VAC-COVID-19-Scale-negative subscale	r	−0.203	−0.127	0.537	0.481	0.360	0.428	0.275		
	*p*	0.022 *	0.155	<0.001 *	<0.001 *	<0.001 *	<0.001 *	0.002 *		
VAC-COVID-19-Scale-positive subscale	r	−0.152	0.089	0.555	0.488	0.341	0.452	0.273	0.466	
	*p*	0.088	0.319	<0.001 *	<0.001 *	<0.001 *	<0.001 *	0.002 *	<0.001 *	
VAC-COVID-19-Scale-total score	r	−0.224	−0.019	0.612	0.529	0.408	0.486	0.301	0.829	0.843
	*p*	0.011 *	0.831	<0.001 *	<0.001 *	<0.001 *	<0.001 *	0.001 *	<0.001 *	<0.001 *

Abbreviations: A—DrVac-COVID19S autonomy subscale; CAS—Coronavirus Anxiety Scale; DrVac-COVID19S—The Drivers of COVID-19 Vaccination Acceptance Scale; FCV-19S—The Fear of COVID-19 Scale; I—DrVac-COVID19S impacts subscale; K—DrVac-COVID19S knowledge subscale; *p*—*p*-value, r—Spearman’s rank correlation coefficient, V—DrVac-COVID19S values subscale; VAC-COVID-19—Scale to Measure the Perception of SARS-CoV-2 Vaccines Acceptance, and *—statistically significant.

## Data Availability

Data are available upon reasonable request.
